# Folliculin: A Regulator of Transcription Through AMPK and mTOR Signaling Pathways

**DOI:** 10.3389/fcell.2021.667311

**Published:** 2021-04-26

**Authors:** Josué M. J. Ramirez Reyes, Rafael Cuesta, Arnim Pause

**Affiliations:** ^1^Goodman Cancer Research Center, McGill University, Montréal, QC, Canada; ^2^Department of Biochemistry, McGill University, Montréal, QC, Canada

**Keywords:** folliculin, mTORC1, AMPK, TFE3, TFEB, PGC1α, transcriptional regulation, metabolism

## Abstract

*Folliculin (FLCN)* is a tumor suppressor gene responsible for the inherited Birt-Hogg-Dubé (BHD) syndrome, which affects kidneys, skin and lungs. FLCN is a highly conserved protein that forms a complex with folliculin interacting proteins 1 and 2 (FNIP1/2). Although its sequence does not show homology to known functional domains, structural studies have determined a role of FLCN as a GTPase activating protein (GAP) for small GTPases such as Rag GTPases. FLCN GAP activity on the Rags is required for the recruitment of mTORC1 and the transcriptional factors TFEB and TFE3 on the lysosome, where mTORC1 phosphorylates and inactivates these factors. TFEB/TFE3 are master regulators of lysosomal biogenesis and function, and autophagy. By this mechanism, FLCN/FNIP complex participates in the control of metabolic processes. AMPK, a key regulator of catabolism, interacts with FLCN/FNIP complex. FLCN loss results in constitutive activation of AMPK, which suggests an additional mechanism by which FLCN/FNIP may control metabolism. AMPK regulates the expression and activity of the transcriptional cofactors PGC1α/β, implicated in the control of mitochondrial biogenesis and oxidative metabolism. In this review, we summarize our current knowledge of the interplay between mTORC1, FLCN/FNIP, and AMPK and their implications in the control of cellular homeostasis through the transcriptional activity of TFEB/TFE3 and PGC1α/β. Other pathways and cellular processes regulated by FLCN will be briefly discussed.

## Introduction

*Folliculin* (*FLCN*) gene was first identified in 2002 as the gene responsible for the *Birt-Hogg-Dubé* (BHD) syndrome (Birt et al., 1977; Nickerson et al., 2002). BHD is an autosomal dominant inherited disorder characterized by the presence of fibrofolliculomas, lung cysts, an increased frequency of spontaneous pneumothorax, and bilateral multifocal renal tumors (Birt et al., 1977; Schmidt and Linehan, 2018). Most of germline mutations in *FLCN* gene are frameshift or splice site mutations predicted to produce unfunctional truncated FLCN proteins (Schmidt and Linehan, 2018). Rarer BHD-linked *FLCN* mutations result in the expression of missense and short in-frame deletions variants. Recent studies have shown that the loss-of-function associated with these variants is due to proteasomal degradation of FLCN proteins (Nahorski et al., 2011; Clausen et al., 2020). FLCN is considered a tumor suppressor, following the Knudson “second hit” model, since somatic mutation or loss of heterozygosity of the non-affected allele has been observed in BHD-associated renal tumors and in animal models (Schmidt and Linehan, 2018).

**FIGURE 1:**
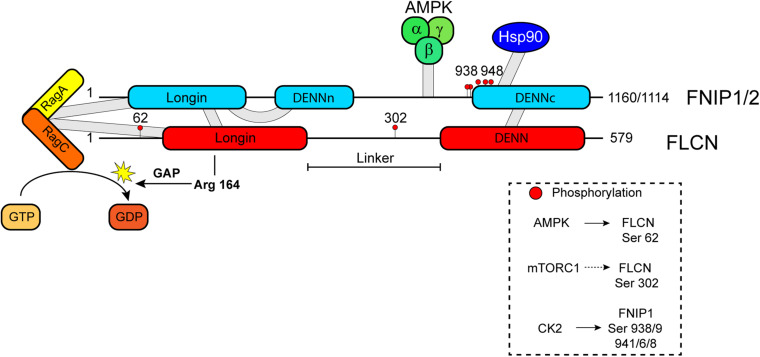
Schematic diagram of FLCN and FNIP domains and their interacting partners AMPK, Hsp90, and Rag A/C. FLCN GAP activity on Rag C is indicated as well as the catalytic arginine finger Arg 164. Phosphorylation sites within FLCN and FNIP sequences are marked by red-filled cycles and putative (dashed arrow) or known kinases (continuous arrow) are indicated in the box.

Human FLCN is a protein of 579 amino acids highly conserved across species (de Martin Garrido and Aylett, 2020). FLCN does not show sequence homology to known proteins or well-defined functional domains. However, the three-dimensional structure of FLCN shares structural similarity with DENN1B protein. Accordingly, it was predicted that FLCN contains a *differentially expressed in normal and neoplasia* (DENN) domain at its C-terminus and an N-terminal Longin domain (Figure 1; Wu et al., 2011; Nookala et al., 2012). The proteins with these structural domains have been characterized as *guanine nucleotide exchange factors* (GEF) for Rab GTPases (Yoshimura et al., 2010), promoting their activity in membrane trafficking. However, FLCN GEF activity was only detected on Rab35 in *in vitro* assays (Nookala et al., 2012). In contrast, FLCN shows *GTPase activating protein* (GAP) activity for Rag C/D and Rab7A small GTPases. Consequently, FLCN has been implicated in lysosomal mTORC1 recruitment and activation, endocytic trafficking of *epidermal growth factor receptor* (EGFR), and intracellular distribution of the lysosomes (Schmidt and Linehan, 2018; de Martin Garrido and Aylett, 2020) (see below).

*Folliculin* forms complexes with two proteins, *folliculin interacting protein 1* (FNIP1) and *folliculin interacting protein 2* (FNIP2) (Baba et al., 2006; Hasumi et al., 2008; Takagi et al., 2008). A similar complex was detected in yeast, where FNIP ortholog *lethal with sec thirteen 4* (Lst4) interacts with FLCN ortholog Lst7 (Pacitto et al., 2015). FNIP2 shows high sequence similarity to FNIP1, and both proteins are conserved across species. Like FLCN, FNIP1 and FNIP2 contain an N-terminal Longin domain and a C-terminal DENN domain (Zhang et al., 2012). Initial binding studies determined the interaction of FNIP1/2 with the C-terminal region of FLCN (Baba et al., 2006; Hasumi et al., 2008; Takagi et al., 2008), but recent structural analyses propose the formation of functional FLCN/FNIP complexes by heterodimerization through their Longin and DENN domains (Figure 1; Lawrence et al., 2019; Shen et al., 2019). As functional partners of FLCN, double inactivation of FNIP1/2 in murine kidney produces enlarged polycystic kidneys similar to FLCN deficiency. Additionally, FNIP1 and FNP2 act as tumor suppressors too, since FNIP1 and/or FNIP2 knockout mice exhibit tumors in multiple organs (Hasumi et al., 2015). As expected, a role of FLCN/FNIP complexes in the regulation of Rab GTPases and membrane trafficking has been described (Dodding, 2017). Although initial studies focused on deciphering how FLCN loss results in BHD-associated renal tumors, evidence accumulated during the last decade indicate that FLCN is a pleiotropic protein implicated in multiple cellular processes. The functions of FLCN/FNIP complex mostly occur through the modulation of the activity of two key protein kinases, *mechanistic target of rapamycin complex 1* (mTORC1) and *AMP-activated protein kinase* (AMPK).

Mechanistic target of rapamycin complex 1 and AMPK are two serine/threonine protein kinases with opposite roles in cellular metabolism. mTORC1 is active when the cell is under optimal energetic conditions to grow and proliferate, while AMPK is activated under starvation conditions when AMP levels inside the cell rise (Gonzalez et al., 2020). mTORC1 has been widely described as a positive regulator of anabolic processes such as protein, lipid, and nucleotide synthesis, required for cellular growth and proliferation, and as a negative regulator of autophagy (Gonzalez et al., 2020; Liu and Sabatini, 2020). On the other hand, AMPK inhibits anabolism to minimize ATP consumption and stimulates catabolic processes to increase ATP production (Herzig and Shaw, 2018; Gonzalez et al., 2020). An appropriate balance between these two pathways is critical to ensure homeostasis, and FLCN/FNIP complex contributes to this equilibrium by coordinating mTORC1 and AMPK activities. Additionally, crosstalk between these pathways further coordinates the metabolic adaptation to nutritional and energetic conditions. In this review, we discuss the role of FLCN/FNIP complex as a modulator of mTORC1 and AMPK pathways, emphasizing its effect on the function of downstream *transcription factor binding to IGHM enhancer B* (TFEB) and TFE3, and transcriptional coactivator *peroxisome proliferator-activated receptor-*γ *coactivator* 1α (PGC1α). We additionally review other cellular processes associated with FLCN function.

## Regulation of mTORC1 and AMPK Pathways by FLCN

### Activation of mTORC1 at the Lysosome

Mechanistic target of rapamycin (mTOR) is a 289 kDa serine/threonine protein kinase, member of the *PI3K-related protein kinase* (PIKK) family (Keith and Schreiber, 1995). It is well-conserved in all eukaryotic organisms. mTOR constitutes the catalytic subunit of two different complexes named *mTOR complex 1* (mTORC1) and *mTOR complex 2* (mTORC2). In addition to mTOR, mTORC1 consists of *regulatory-associated protein of mTOR* (RAPTOR) (Hara et al., 2002; Kim et al., 2002) and *mammalian lethal with SEC13 protein 8* (mLST8) (Kim et al., 2003). *Proline-rich AKT substrate 40 kDa* (PRAS40) (Sancak et al., 2007; Vander Haar et al., 2007) and *DEP-domain-containing mTOR-interacting protein* (DEPTOR) (Peterson et al., 2009) are two accessory components of mTORC1 with an inhibitory effect on mTORC1 activity. RAPTOR is a scaffold protein that plays a key role in the localization of mTORC1 at the lysosome for activation (Sancak et al., 2008) and in the recruitment of several mTORC1 substrates such as S6K and 4E-BP1 by binding to their TOR signaling (TOS) motifs (Nojima et al., 2003; Schalm et al., 2003). mTORC1 integrates environmental cues such as nutrients and growth factors as well as the energetic state of the cell to control cell growth and proliferation. Therefore, deregulation of mTORC1 has been associated with several cancer types, metabolic syndromes, and neurodevelopmental disorders (Liu and Sabatini, 2020). mTORC2 is formed by mTOR, mLST8, *rapamycin-insensitive companion of mTOR* (RICTOR), and *stress-activated MAP kinase interacting protein 1* (mSIN1) (Oh and Jacinto, 2011). *Protein associated with RICTOR 1 or 2* (Protor 1/2) (Pearce et al., 2007; Woo et al., 2007) and the inhibitory factor DEPTOR (Peterson et al., 2009) have been also identified as components of mTORC2 complex. mTORC2 regulates cell survival, metabolism, and cytoskeletal remodeling.

Activation of mTORC1 occurs when the levels of nutrients, growth factors, and energy are optimal to support cell growth and proliferation. Sensing systems are required to monitor and integrate these inputs, and trigger mTORC1 signaling. This depends on the nucleotide-loading state of two set of small GTPases, *Ras homolog enriched in brain* (Rheb) (Inoki et al., 2003; Long et al., 2005) and *Ras-related GTP-binding protein* (Rag) GTPases (Kim et al., 2008; Sancak et al., 2008). When amino acid, glucose, and cholesterol are available, active Rag GTPases recruit mTORC1 to the lysosomal surface, where it co-localizes with growth factor-activated GTP-bound Rheb GTPase and is activated. The *tuberous sclerosis complex* (TSC) composed of TSC1, TSC2, and TBC1D7 is a negative regulator of Rheb (Tee et al., 2003b; Dibble et al., 2012). TSC acts as a GAP and promotes the conversion from active GTP-bound to inactive GDP-bound Rheb (Inoki et al., 2003; Tee et al., 2003b). Growth factors activate the serine/threonine kinase Akt, which phosphorylates TSC2 at multiple sites to prevent TSC inactivating effect on Rheb (Inoki et al., 2002; Tee et al., 2003a). Inhibitory phosphorylation of TSC by ERK and p90 ribosomal S6 kinase (RSK) has been also described (Roux et al., 2004; Ma et al., 2005). As mentioned, nutrient-induced activation of Rag GTPases promotes the recruitment of mTORC1 to the lysosomal surface in mammalian and yeast cells. Rag GTPases form heterodimers that consist of two functionally equivalent pairs, Rag A or Rag B in complex with Rag C or Rag D. Rag GTPases are anchored to the lysosomes through their interaction with the pentameric Ragulator complex (Sancak et al., 2010). In their active state, Rag A/B is bound to GTP and Rag C/D is bound to GDP, while in their inactive state Rag A/B is bound to GDP and Rag C/D is bound to GTP. Rag GTPase state is mostly determined by the GAP activity of GATOR1 and FLCN/FNIP toward Rag A/B and Rag C/D, respectively, in response to nutrient availability.

**FIGURE 2:**
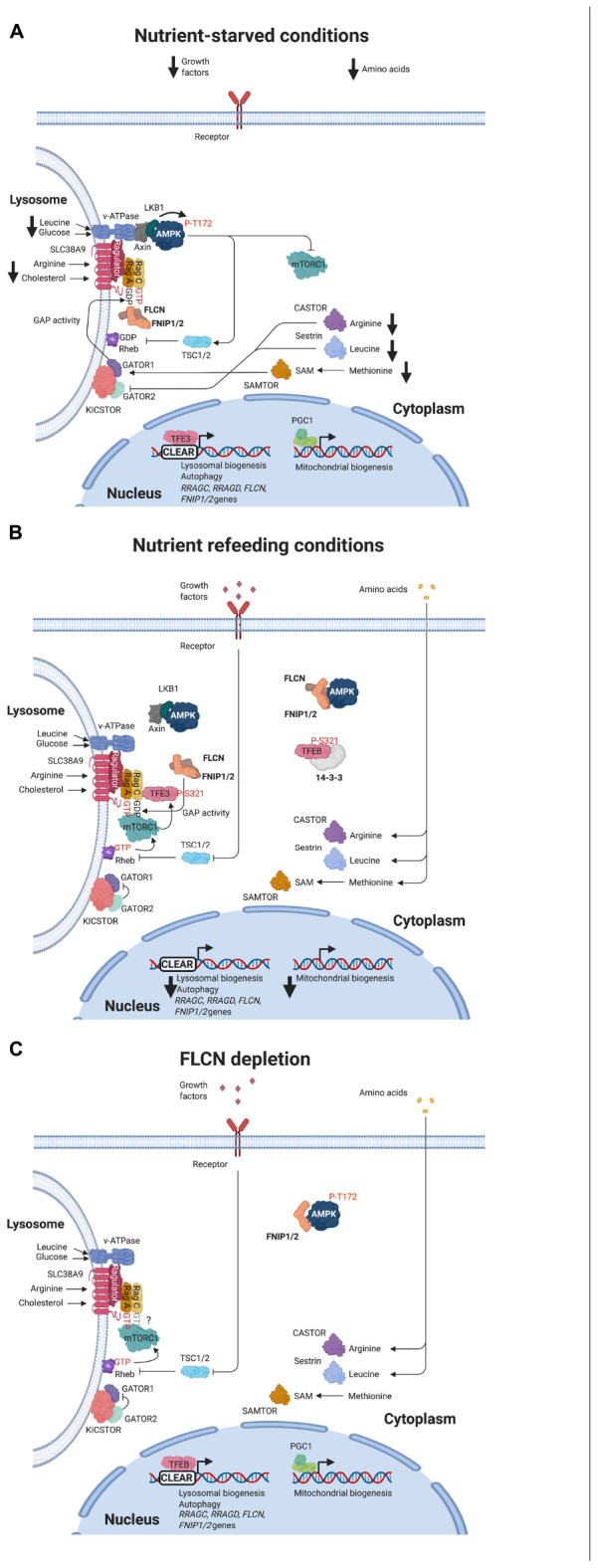
Regulation of mTORC1 and AMPK signaling pathways in response to nutrient availability or FLCN depletion. **(A)** Under nutrient starvation, amino acid sensors facilitate activation of GATOR1 complex, which promotes GTP hydrolysis by Rag A/B. Inactivation of Rag A/B impairs the recruitment of mTORC1 but facilitates the binding of FLCN/FNIP complex to the Rags. Low glucose levels trigger the assembly of AXIN, LKB1, and AMPK with v-ATPase forming a complex that induces LKB1-mediated phosphorylation and activation of AMPK. TFEB/TFE3 localize in the nucleus and transcribe their target genes. PGC1α/β are expressed and active. **(B)** In response to nutrient refeeding, FLCN/FNIP GAP activity on Rag C/D and spontaneous exchange of GDP to GTP on Rag A/B convert Rags to their active state. mTORC1 is recruited and active on the lysosomal surface, where phosphorylates and inactivates TFEB/TFE3. AMPK is inactive. **(C)** FLCN depletion keeps Rag C/D in their inactive state (GTP-bound), which prevents TFEB/TFE3 binding and phosphorylation. These transcription factors translocate to the nuclear and induce the expression of their target genes. PGC1α/β are expressed and active. AMPK is constitutively active.

Cellular levels of specific amino acids such as leucine, arginine, and methionine are sensed by protein complexes, which communicate with the GAP factors that determine Rag GTPase nucleotide state. Among these metabolic sensors, Sestrin2, CASTOR1, and SAMTOR bind to cytoplasmic leucine, arginine, and S-adenosylmethionine, respectively, to modulate GATOR1 GAP activity (Chantranupong et al., 2016; Wolfson et al., 2016; Gu et al., 2017). Under starvation conditions, these sensors interact with GATOR2 complex, a GATOR1 negative regulator, or directly with GATOR1 (for SAMTOR) to facilitate the KICSTOR-mediated recruitment of GATOR1 on the lysosomal surface (Wolfson et al., 2017). At the lysosome, GATOR1 interacts with Rag A/B and promotes GTP hydrolysis, preventing mTORC1 binding (Figure 2A; Bar-Peled et al., 2013; Panchaud et al., 2013). SLC38A9 transmembrane protein is a sensor of lysosomal arginine. In the presence of arginine, the interaction of SLC38A9 with Ragulator-Rag GTPase complex leads to the activation of FLCN/FNIP GAP activity toward Rag C/D (see below), which promotes Rags transition from their inactive to their active state, facilitating mTORC1 recruitment and activation (Figure 2B; Wang et al., 2015; Lawrence et al., 2019; Fromm et al., 2020). SLC38A9 also mediates mTORC1 activation by lysosomal cholesterol (Castellano et al., 2017). *Vacuolar H^+^-adenosine triphosphatase* (v-ATPase) is other sensor of lysosomal amino acids, particularly leucine (Zoncu et al., 2011; Zhang et al., 2014; Milkereit et al., 2015). High levels of leucine induce ATP hydrolysis by v-ATPase, which promotes its binding to Ragulator-Rag GTPase complex to support mTORC1 activation (Figure 2B). v-ATPase is a sensor of the cellular energy status too. Under low glucose conditions, v-ATPase facilitates the assembly of a complex formed by AXIN, *liver kinase B1* (LKB1), AMPK, and Ragulator to prevent mTORC1 activation (Figure 2A; Zhang et al., 2014). This inhibitory effect was proposed to occur through the blocking of Ragulator GEF activity to the Rag A/B and the activation of AMPK by LKB1 (Figure 2A). Nowadays, the role of Ragulator as a GEF for Rag A/B has been questioned (Lawrence et al., 2019). AMPK represses mTORC1 through the phosphorylation of RAPTOR and TSC (see below). v-ATPase senses glucose levels through its interaction with aldolase. In the presence of glucose, the levels of *fructose-1,6-biphosphate* (FBP), an aldolase substrate, are elevated and prevent the binding of aldolase to v-ATPase. When glucose levels drop, aldolase is able to interact with v-ATPase and promote the assembly of AMPK-containing complex (Zhang et al., 2017).

*Folliculin*/*folliculin interacting protein* complex was characterized as a GAP for Rag C/D and, initially, proposed as a positive modulator of mTORC1 (Tsun et al., 2013). Under amino acid starvation, GATOR1-induced inactivation of Rag A/B promotes the binding of FLCN-FNIP to the Rag GTPases on the lysosome (Figure 2A; Petit et al., 2013; Tsun et al., 2013; Meng and Ferguson, 2018). Consequently, FLCN/FNIP form a stable complex with inactive GDP-loaded Rag A/GTP-bound Rag C and Ragulator (Figure 2A; Lawrence et al., 2019). Using cryoelectron microscopy (cryo-EM), it was established that Longin domains of FLCN and FNIP form a heterodimer that contacts both nucleotide binding domains of the Rag heterodimer, while the DENN domains heterodimerize at the distal end of the structure (Lawrence et al., 2019; Shen et al., 2019). It was also resolved the interaction of the C-terminal roadblock domains of the Rags with Ragulator. These structural studies allow identifying Arg164 within the Longin domain of FLCN as the catalytic residue for GAP activity, but this arginine is far from Rag C/D nucleotide pocket, suggesting that FLCN GAP activity is inhibited (Figure 1; Lawrence et al., 2019; Shen et al., 2019). However, it is not clear how this activity is stimulated when amino acids levels are recovered. It has been recently proposed that the binding of SLC38A9 to the Rags may induce a conformational change in FLCN/FNIP complex that inserts Arg164 finger into the nucleotide binding pocket of Rag C/D to stimulate GTP hydrolysis (Shen et al., 2019; Fromm et al., 2020). SLC38A9 dissociation would enable spontaneous GDP to GTP exchange on Rag A to generate an active Rag heterodimer (Fromm et al., 2020). mTORC1 is then recruited to the lysosome through the interaction of RAPTOR with the nucleotide binding domains of Rag heterodimers, and it is allosterically activated by GTP-bound Rheb (Figure 2B; Anandapadamanaban et al., 2019; Rogala et al., 2019). The role of FLCN/FNIP complex as a positive modulator of mTORC1 activity is controversial. While depletion of FLCN in certain cell lines impairs mTORC1 activation as determined by decreased phosphorylation of S6K (Petit et al., 2013; Tsun et al., 2013), FLCN loss does not affect or even increases mTORC1 activity in cells or BHD-derived kidney tumors (Baba et al., 2008; Hasumi et al., 2009; Wada et al., 2016; El-Houjeiri et al., 2019; Napolitano et al., 2020). In fact, hyperactivation of mTORC1 is observed in these tumors. Recent reports support a substrate-specific effect of FLCN GAP on mTORC1 activity. Thus, mTORC1 substrates containing a TOS motif such as S6K and 4E-BP1 require GTP-Rheb, but not GDP-bound Rag C/D, to be phosphorylated on the lysosome. However, mTORC1 substrates without a TOS motif such as the transcription factors TFE3 and TFEB need FLCN GAP activity to interact with the Rags to be phosphorylated (Napolitano et al., 2020). TFEB and TFE3, members of the MiTF/TFE family of transcription factors, are master regulators of autophagy and lysosome biogenesis. Phosphorylation of these factors by mTORC1 prevents their nuclear translocation and transcriptional activity (see below). Consequently, FLCN loss permits nuclear translocation of TFEB/TFE3 and transcription of their target genes (Figure 2C). This group of genes includes *RRAGC* and *RRAGD* that encode for Rag C and Rag D, respectively (Di Malta et al., 2017; Li et al., 2019). Upregulation of these two Rags may explain enhanced activation of mTORC1 associated with FLCN loss in BHD-derived renal tumors (Napolitano et al., 2020).

### FLCN/FNIP Interaction With AMPK

AMP-activated protein kinase is a positive regulator of catabolic metabolism and a negative regulator of anabolic processes under low energy conditions. Therefore, AMPK is an essential player in energetic homeostasis. AMPK is a heterotrimeric complex composed of three subunits: α, β, and γ (Herzig and Shaw, 2018; Gonzalez et al., 2020). The α subunit has the protein kinase catalytic domain of the heterotrimer, the β subunit contains the binding sites for the other subunits, and the γ subunit contains four tandem cystathionine-B-synthase (CBS) domains that bind adenosine nucleotides (Xiao et al., 2013; Calabrese et al., 2014; Gonzalez et al., 2020). In humans, there are two α subunits, α1 and α2, two β subunits, β1 and β2, and three γ subunits, γ1, γ2, and γ3, that can combine to form twelve different AMPK heterotrimers (Ross et al., 2016). Under low energy conditions, AMP/ATP ratio is high and AMP binds to the γ subunit to allosterically activate AMPK. Additionally, AMP stimulates the phosphorylation of the α subunit at Thr172 and prevents its dephosphorylation, which further enhance AMPK activity (Suter et al., 2006; Gowans et al., 2013). Two upstream kinases have been identified as responsible for AMPK phosphorylation at Thr172 *liver kinase B1* (LKB1) and *Ca^2+^/calmodulin-dependent kinase kinase* β (CaMKKβ) (Oakhill et al., 2011; Gowans et al., 2013). In addition to AMP, AMPK can sense other ligands such as glycogen. Binding of glycogen to the β-*carbohydrate-binding module* (β-CBM) in the β subunit inhibits AMPK activity (Polekhina et al., 2003; McBride et al., 2009). As described above, AMPK is activated by LKB1 phosphorylation on the lysosome, where it is recruited by AXIN under low glucose conditions (Zhang et al., 2014, 2017).

AMP-activated protein kinase forms a complex with FNIP1, FNIP2, and FLCN through its binding to the C-terminal region of FNIP1 or FNIP2 (Figure 1; Baba et al., 2006; Hasumi et al., 2008; Takagi et al., 2008), but the functional relevance of this interaction is not known. However, there is genetic evidence that support a role of FLCN/FNIP complex as a negative regulator of AMPK. Thus, depletion of FLCN or FNIP1 results in constitutive activation of AMPK in *C. elegans* and mammalian cells (Possik et al., 2014, 2015; Yan et al., 2014; Siggs et al., 2016; El-Houjeiri et al., 2019). Loss of FLCN leads to AMPK-dependent increase in autophagy, HIF-1/2 activity, and resistance to obesity, energy stress, osmotic stress, and pathogens. How FLCN/FNIP complex controls AMPK activity needs to be elucidated. To solve this question, it would be important to determine whether FLCN/FNIP binding and/or its GAP activity is required to modulate AMPK function. Regarding its GAP activity, AMPK is a substrate for mTORC1 or S6K. Recently, it has been described that phosphorylation of AMPK at α1 Ser347 or α2 Ser345 by mTORC1 decreases phosphorylation at Thr172 (Ling et al., 2020). Similarly, phosphorylation of AMPK α2 subunit at Ser491 by S6K reduces AMPK activity (Dagon et al., 2012). Accordingly, inhibition of mTORC1 results in increased phosphorylation of AMPK at Thr172 and elevated AMPK activity (Ling et al., 2020). Based on these results, it is possible to speculate that, upon FLCN or FNIP loss, reduced mTORC1 activity may trigger AMPK activation in certain cellular contexts. In other cellular settings such as FLCN-null human renal carcinoma cells UOK257 or kidneys from kidney-specific FLCN knockout mice, mTORC1 activity was upregulated as a result of increased expression of Rag C and Rag D GTPases (Di Malta et al., 2017), while no effect on AMPK activity was observed (Baba et al., 2006; Napolitano et al., 2020). The hyperactivation of mTORC1 might prevent AMPK activation in this context. This is an important question to be investigated since it adds a new crosstalk between mTORC1 and AMPK, and positions FLCN/FNIP complex as a key linker of these two metabolic pathways. Nevertheless, the impact of FLCN loss on AMPK signaling needs further investigation since other studies showed reduced AMPK activity in FLCN-null cells. Thus, FLCN knockout affects subcellular localization and expression of LKB1, which leads to reduced AMPK activity in alveolar epithelial cells and ciliated cells (Goncharova et al., 2014; Zhong et al., 2016). Decreased AMPK signaling was also associated with FLCN loss in FLCN-deficient heart (Hasumi et al., 2014). In this case, PGC1α-induced mitochondrial function causes the accumulation of ATP and the subsequent inactivation of AMPK in the absence of FLCN. To add more complexity, mTORC1 and AMPK directly or indirectly phosphorylate FLCN at Ser302 and Ser62, respectively (Figure 1; Piao et al., 2009; Wang et al., 2010; Zoncu et al., 2011). The effect of these modifications on FLCN function are not well-characterized, but phosphorylation at Ser62 increases FLCN binding to AMPK, while phosphorylation at Ser302 has the opposite effect. None of them alters the interaction of FLCN with FNIP. AMPK also phosphorylates FNIP (Baba et al., 2006). Additionally, AMPK controls FLCN expression at transcriptional level through TFEB (Collodet et al., 2019). These results underline the presence of a complex interplay between mTORC1, AMPK, and FLCN/FNIP that need more in-depth studies to be elucidated.

AMP-activated protein kinase in turn regulates mTORC1 through the phosphorylation of TSC2 and RAPTOR (Inoki et al., 2002; Gwinn et al., 2008). When active, AMPK phosphorylates TSC2 at Thr1271 and Thr1387, which activates its GAP activity for Rheb and blocks growth factor-induced mTORC1 stimulation. Additionally, phosphorylation of RAPTOR at Ser722 and Ser792 promotes its association with 14-3-3 and, therefore, impairs the recruitment of mTORC1 to the Rags. All these mechanisms and crosstalk remark the importance of a coordinated and regulated response to environmental and nutritional changes to preserve cellular homeostasis, as well as the critical function of mTORC1-FLCN/FNIP-AMPK axis in these processes.

## Transcriptional Regulation of Metabolism by mTORC1-FLCN/FNIP-AMPK Axis: PGC1α and Tfeb/Tfe3

### mTOR and TFEB/TFE3 Axis

Transcription factor binding to IGHM enhancer B and TFE3 are members of the MiTF family of helix-loop-helix leucine zipper transcriptional factors, which also includes MiTF and TFEC (Hemesath et al., 1994). There are homologous genes in *Drosophila*, *C. elegans*, and *Saccharomyces cerevisiae*, suggesting they play a fundamental role through evolution (Rehli et al., 1999; Hallsson et al., 2004; Lapierre et al., 2013; Srivas et al., 2016). They bind to the DNA sequence known as *Coordinated Lysosomal Expression and Regulation* (CLEAR) motif through their basic helix-loop-helix domain (Sardiello et al., 2009). For DNA binding, they may form homo or heterodimers with other members of the family via the leucine zipper (Pogenberg et al., 2012). These transcriptional factors regulate the expression of genes involved in lysosomal biogenesis, autophagy, lipid metabolism, and immune and stress responses (Settembre et al., 2011, 2013; Martina et al., 2014; El-Houjeiri et al., 2019). Because of their implication in multiple cellular processes, dysregulation of these factors has been associated with cancer, metabolic syndromes, and neurological disorders. Thus, chromosomal translocation of *TFE3* and *TFEB* genes are detected in renal cell carcinoma, alveolar soft part sarcoma, and perivascular epithelioid cell neoplasm (Argani et al., 2010a; Hodge et al., 2014; Kauffman et al., 2014; Perera et al., 2019). Additionally, activation of TFE3 and TFEB is observed in other cancer types such as lung, pancreatic ductal adenocarcinoma, breast, prostate, and colorectal (Liang et al., 2018; Torres et al., 2018; Perera et al., 2019) (see below). These factors have also been associated with lysosomal storage diseases. Moreover, defective autophagic flux caused by low levels of TFEB has been linked to the development of Alzheimer’s, Parkinson’s, and Huntington’s diseases (Raben and Puertollano, 2016).

As described above, subcellular localization of TFEB and TFE3 is controlled by mTORC1. When amino acids are available, TFEB and TFE3 are recruited to the lysosomal membrane by their binding to active GDP-loaded Rag C/D. This interaction requires a sequence of 30 amino acids localized at the N-terminal of both TFEB (aa 1-30) and TFE3 (aa 106-135) (Martina and Puertollano, 2013). Concomitantly, mTORC1-mediated phosphorylation of TFEB at the Ser211 or TFE3 at Ser321 promotes their interaction with 14-3-3 and their retention in the cytoplasm (Figure 2B; Martina et al., 2012; Roczniak-Ferguson et al., 2012; Settembre et al., 2012; Martina et al., 2014). Under nutrient-starvation conditions or FLCN loss, TFEB and TFE3 do not interact with inactive Rags and translocate to the nucleus, where they activate their target genes (Figures 2A,C; Hong et al., 2010a; Martina et al., 2012, 2014; Roczniak-Ferguson et al., 2012; Settembre et al., 2012). In addition to mTORC1, other pathways regulate TFEB and TFE3 subcellular localization and function. Hence, phosphorylation of TFEB at Ser142 by *extracellular signal-regulated kinase 2* (Erk2), at Ser467 by Akt, or at Tyr173 by c-Abl prevents its nuclear translocation (Settembre et al., 2011; Palmieri et al., 2017; Contreras et al., 2020). Similarly, phosphorylation of TFEB at Ser3 by *mitogen-activated protein kinase kinase kinase kinase 3* (MAP4K3), or at Ser134 and Ser138 by *glycogen synthase kinase* 3β (GSK3β) contributes to TFEB cytoplasmic retention by promoting its binding to the Rag GTPases-Ragulator complex and, therefore, its phosphorylation by mTORC1 (Li et al., 2016; Hsu et al., 2018). In contrast, AMPK induces nuclear activation of TFEB/TFE3 under nutrient starvation conditions or in FLCN deficient cells (Eichner et al., 2019; El-Houjeiri et al., 2019; Paquette et al., 2021). However, the mechanisms by which these pathways regulate TFEB/TFE3 are not fully characterized. In addition to TFEB subcellular localization, the phosphorylation of TFEB may determine its stability and activity. Hence, chaperone-dependent E3 ubiquitin ligase *STIP1 homology and U-Box containing protein 1* (STUB1) binds to inactive TFEB phosphorylated at Ser142 and S211 and induces its degradation by the proteasome under stress condition, facilitating nuclear translocation and activity of unphosphorylated TFEB (Sha et al., 2017). In osteoclast, phosphorylation at Ser462, Ser463, Ser467 and SerS469 by protein kinase C β (PKCβ) stabilizes and activates TFEB transcriptional activity (Ferron et al., 2013). Similarly, phosphorylation of TFEB and TFE3 at the C-terminal region (Ser466, Ser467 and Ser 469 in TFEB) by AMPK increases their transcriptional activity under starvation conditions, FLCN loss, or pharmacological inhibition of mTORC1 or activation of AMPK in MEFs (Paquette et al., 2021).

Appropriate balance between mTORC1 and AMPK pathways in response to nutritional and environmental inputs is critical to maintain cellular homeostasis. Therefore, crosstalk between these two pathways has been described at different levels. Similarly, TFEB and TFE3 trigger feedback loops to activate mTORC1. These mechanisms involve TFEB/TFE3-mediated transcriptional upregulation of the expression of Rag C and Rag D as well as FLCN and FNIP1/2 under starvation conditions (Martina et al., 2014; Di Malta et al., 2017; Li et al., 2019; Endoh et al., 2020; Napolitano et al., 2020). Accordingly, CLEAR sequences have been identified at the promoters of these genes. By increasing the amount of Rag C/D and/or FLCN/FNIP, TFEB and TFE3 facilitate a rapid reactivation of mTORC1 when nutrient levels are restored. In turn, this would result in TFEB/TFE3 cytoplasmic sequestration and inactivation. In FLCN deficient cells, upregulation of Rag C and Rag D may drive mTORC1 hyperactivation without affecting TFEB and TFE3 localization or activation.

### AMPK-PGC1α Axis

The PGC1 family is composed of three members: PGC1α, PGC1β, and PRC (Villena, 2015). All of them are transcriptional coactivators involved in mitochondrial biogenesis and oxidative metabolism. They are mainly present in tissues with high metabolic demands such as muscle, heart, kidney, and brown adipose tissue (Villena, 2015). However, their activity depends on the stimuli and the cellular context (Lin et al., 2005). They were first characterized as cofactors for the *peroxisome proliferator-activated receptor-*γ (PPAR-γ). Nevertheless, they interact with over twenty different transcription factors and increase their activity (Chambers and Wingert, 2020), suggesting a wide role of PGC coactivators in the regulation of gene expression. In addition to control mitochondrial biogenesis and oxidative metabolism, PGC1α is involved in gluconeogenesis, fatty acid oxidation, and glucose transport (Wu et al., 1999; Villena, 2015). More recent studies have expanded the function of these coactivators to other cellular processes. From genome-wide DNA binding and gene expression profiles in loss- or gain-of-function models, PGCs have been implicated in phospholipid biosynthesis, angiogenesis, glycogen metabolism, autophagy, protection against oxidative stress, muscle fiber specification, and immune response (Villena, 2015; Chang et al., 2018).

PGC1α contains an N-terminal activation domain, a central regulatory domain, and a C-terminal RNA recognition motif (Lin et al., 2005; Villena, 2015). The activation domain recruits *histone acetyltransferase* (HAT) proteins such as SRC-1 and CBP/p300 in order to induce chromatin remodeling, since PGC1α lacks this enzymatic activity (Puigserver et al., 1999). The central domain contains leucine-rich motifs important for its binding to partner transcription factors. C-terminal RNA recognition motif facilitates the association with proteins of the mediator complex that interacts with the RNA polymerase II machinery (Wallberg et al., 2003). Additionally, this region harbors a Ser/Arg-rich domain and an RNA binding domain that have been associated with the coupling of pre-mRNA splicing with transcription (Monsalve et al., 2000).

Although PGC1α expression is upregulated in response to exercise, contractile stimulation *in vitro*, fasting, or cold exposure (Philp et al., 2011; Rowe et al., 2012; Villena, 2015), PGC1α activity is also modulated by post-translational modifications. These modifications include phosphorylation, methylation, acetylation, and deacetylation (Fernandez-Marcos and Auwerx, 2011; Villena, 2015). Most of them modulate PGC1α activity, although phosphorylation may control PGC1α protein stability. Thus, its phosphorylation by p38 mitogen-activated protein kinase in response to cytokines prevents PGC1α degradation by the proteasome (Puigserver et al., 2001; Trausch-Azar et al., 2010), which results in enhanced activity. In contrast, GSK-3β-mediated phosphorylation promotes its degradation (Anderson et al., 2008).

AMP-activated protein kinase is other well-known regulator of PGC1α that tightly controls its expression and activity. Thus, exercise- or AICAR-induced activation of AMPK leads to transcriptional upregulation of PGC cofactors in skeletal muscle (Terada and Tabata, 2004; Lee et al., 2006). However, how this regulation occurs remain elusive. A possible mechanism involves TFEB and TFE3, which promote transcription of PGC1α and PGC1β in different cellular settings (Settembre et al., 2013; Salma et al., 2015; Wada et al., 2016; Baba et al., 2018). Supporting this possibility, AMPK-mediated activation of TFEB and TFE3 has been reported (Eichner et al., 2019; El-Houjeiri et al., 2019). Nevertheless, this model needs to be experimentally validated. Additionally, AMPK controls PGC1α activity through the phosphorylation of Ser177 and Ser538. These modifications lead to the activation of PGC1α transcriptional program in skeletal muscle (Jager et al., 2007).

### Transcriptional Regulation by FLCN, AMPK and mTOR

In this section, we will describe cellular processes regulated by the mTORC1-FLCN/FNIP-AMPK axis through TFEB/TFE3 and/or PGC cofactors. We focus on the contribution of FLCN as a modulator of mTORC1-mediated regulation of TFEB/TFE3 activity to metabolic processes such as lysosomal activity, autophagy, and mitochondrial biogenesis. Similarly, we will discuss the function of FLCN as a negative regulator of AMPK and its effect on the transcriptional activity of PGC1α/β in the kidney, adipose tissue, heart, skeletal muscle, and osteoclastogenesis. An interplay between TFEB/TFE3 and PGC1α/β has been described, and we will discuss how this positive feedback loop contributes to their transcriptional function in different cellular processes. Additionally, we review the role of these pathways in tumorigenesis and stem cell differentiation.

#### FLCN/AMPK/PGC1α in Mitochondrial Biogenesis

**FIGURE 3:**
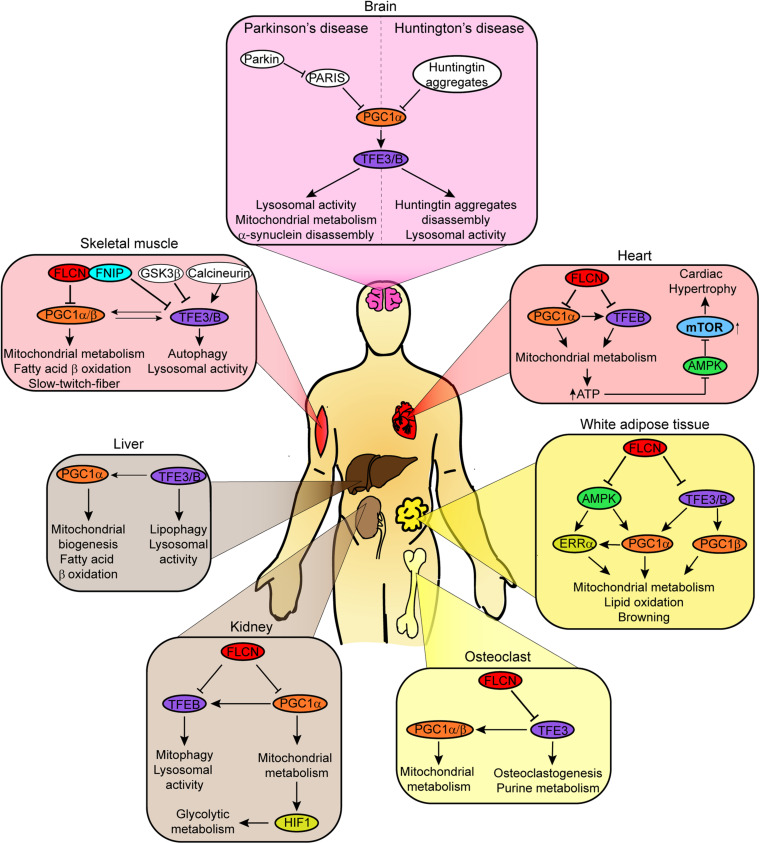
Schematic diagram shows tissue-specific effect of FLCN depletion on the transcriptional activity of TFEB/TFE3 and/or PGC1α/β. The metabolic processes affected by the specific regulatory mechanisms are indicated. AMPK and mTORC1 are only shown when mentioned in the corresponding references.

As a tumor suppressor, FLCN loss induces a polycystic phenotype in the kidney and the development of cystic renal cell carcinoma (Baba et al., 2008; Chen et al., 2008). This phenotype was posteriorly associated with a significant increase in mitochondrial biogenesis (Hasumi et al., 2012). Using a FLCN-deficient kidney model, it was found that FLCN loss leads to increased PGC1α expression, which results in elevated mitochondrial function and oxidative metabolism in renal cancer cells (Figure 3). FLCN reconstitution or elimination of PGC1α was enough to rescue the phenotype associated with FLCN-deficiency (Hasumi et al., 2012). PGC1α upregulation associated with FLCN loss could be mediated by AMPK, but this hypothesis needs further investigation to be confirmed.

Our group has previously shown that FLCN controls oxidative phosphorylation, fatty acid metabolism, and adipogenesis in *white adipose tissue* (WAT) via AMPK/PGC1α/ERRα axis (Yan et al., 2016). FLCN deficiency in WAT protects mice from diet-induced obesity and increases resistance to cold exposure. These physiological changes were linked to elevated AMPK activity, which leads to upregulation of PGC1α and *estrogen-related receptor* α (ERRα) expression. Transcriptional activity of ERRα, an orphan nuclear receptor, is activated by PGC1α (Schreiber et al., 2003; Kallen et al., 2004). Consequently, expression of genes involved in mitochondrial metabolism (*Cox5b, Cox8b, Ndufb4, Uqcr10, Tfam*, and *Mterf1*), lipid oxidation (*Acadm*), and browning markers (*Ucp1, Ucp3, Prdm16, Cidea* and *Dio3*) was increased (Figure 3). Similar metabolic reprogramming was observed *in vitro* as well (Yan et al., 2016), confirming that the absence of FLCN enhances oxidative phosphorylation and browning in WAT (Yan et al., 2016).

Similarly, FLCN or FNIP1 loss promotes PGC1α/β-dependent expression of genes involved in mitochondrial metabolism in skeletal muscle, resulting in metabolic reprogramming (Hasumi et al., 2012; Reyes et al., 2015). Thus, upregulation of genes expressing mitochondria components (*Atp5g, Cox5a, Cycs, Pdk4, Ndufs8* and *Ucp3*) as well as components of the *electron transport chain* (ETC), *tricarboxylic acid cycle* (TCA), and fatty acid-β oxidation is observed in FLCN/FNIP-deficient skeletal muscle. As a result, there is a switch toward oxidative metabolism, increased ATP levels, and differentiation to slow-twitch-fibers (Figure 3; Lin et al., 2002; Reyes et al., 2015; Zhou et al., 2017). This phenotype was linked to PGC1α/β expression, since depletion of these cofactors reversed it. In skeletal muscle, there are sufficient evidence to support a key role of active AMPK in the control of PGC1α/β expression and function (Jager et al., 2007; Reyes et al., 2015; Zhou et al., 2017). Thus, it is probable that AMPK mediates PGC1α/β activation in response to FLCN/FNIP loss in muscle.

Like in other high energy demanding tissues, PGC1α has an important role in mitochondrial biogenesis in the heart (Lehman et al., 2000). Hence, increased expression of PGC1α in FLCN-deficient cardiomyocytes results in upregulation of genes involved in mitochondrial biogenesis and metabolism (Hasumi et al., 2014). Consequently, elevated oxidative metabolism produces sufficient ATP to fulfill high energy demand of the heart. Unlike in other tissues, AMPK remains inactive in FLCN-deficient heart due to the high concentration of ATP. Reduced AMPK activity was associated with hyperactivation of mTORC1 and cardiac hypertrophy, which is prevented by rapamycin (Figure 3; Hasumi et al., 2014). In this context, PGC1α activation occurs independently of AMPK.

Osteoclast differentiation or osteoclastogenesis is a process that relies on mitochondrial metabolism to obtain the high levels of energy required (Lemma et al., 2016). As a master regulator of mitochondrial biogenesis, PGC1β plays a critical role (Ishii et al., 2009). During osteoclastogenesis, FLCN deficiency promotes the expression of *Nfactc1*, a key transcriptional factor for this process (Takayanagi et al., 2002), and the nuclear translocation and activation of TFE3 (Baba et al., 2018). TFE3 induces the expression of PGC1α/β, markers of osteoclast differentiation, and enzymes involved in purine metabolism. PGC1β, but not PGC1α, promotes the transcription of mitochondrial metabolism related genes. Accumulation of ATP and purinergic metabolites accelerate a purinergic signal loop and enhance osteoclastogenesis (Figure 3; Baba et al., 2018). The role of AMPK in osteoclast differentiation needs to be investigated.

Although a lot of evidence underlines the role AMPK as a transcriptional regulator through the control of PGC cofactors, there are still multiple questions to be answered about how this regulation occurs, particularly in FLCN-deficient settings. First, it is necessary to understand how FLCN/FNIP complex regulates AMPK activity. Additionally, it needs to be elucidated the mechanisms by which PGC1α/β expression is enhanced by AMPK in the absence of FLCN. Other question to address is the identification of the transcription factors involved in this regulatory pathway. Our group characterized ERRα as the transcriptional factor that cooperates with PGC1α to control the transcription of target genes downstream of AMPK in WAT (Yan et al., 2016). However, there is not much known about other transcription factors. All these questions need to be investigated in the coming years to fully understand regulation of transcription through the FLCN/AMPK/PGC axis.

#### TFEB/3-PGC1α Crosstalk

Although we have separately discussed the functions of AMPK-PGC1α and mTOR-TFEB/TFE3 axis in transcriptional regulation, several reports support coordinated activity of TFEB/TFE3 and PGC1α on the transcription of specific sets of genes.

In skeletal muscle, TFEB is upregulated and translocates to the nucleus in response to acute contractile activity or exercise (Mansueto et al., 2017; Erlich et al., 2018). TFEB nuclear localization required its dephosphorylation by calcium-stimulated calcineurin phosphatase (Medina et al., 2015). Once in the nucleus, TFEB promotes the expression of genes involved in lysosomal and mitochondrial biogenesis and autophagy. How TFEB upregulates mitochondrial genes is controversial, since both PGC1α-dependent (Erlich et al., 2018) and PGC1α-independent mechanism have been proposed (Mansueto et al., 2017). Additionally, increased TFEB expression may require PGC1α activity, since PGC1α depletion reduces TFEB promoter activity and the expression of mitochondrial genes. These results suggest the existence of a positive feedback loop between TFEB and PGC1α to coordinately control mitochondrial biogenesis and activity (Erlich et al., 2018). This mechanism may contribute to maintain a healthy mitochondrial network, since TFEB, in a PGC1α-dependent or -independent way, promotes the mitochondrial biogenesis, while, by inducing lysosome activity and autophagy, eliminates dysfunctional mitochondria (Figure 3; Erlich et al., 2018). Mitophagy upregulation by TFEB is arguable, since other studies do not observe any effect of TFEB overexpression or depletion on autophagy flux (Erlich et al., 2018). TFE3 may be involved in this regulatory mechanism too, since PGC1α depletion in mice reduces TFE3 expression, and TFE3 controls PGC1α transcription in myotubes (Salma et al., 2015; Erlich et al., 2018). To solve the discrepancies, it would be necessary to determine the contribution of TFEB and TFE3 to the metabolic adaptation of skeletal muscle to exercise. AMPK may be an upstream regulator of TFEB/TFE3 and PGC1α in skeletal muscle, since it is upregulated by exercise and has been previously implicated in the control of these factors. A potential negative regulator may be *glycogen synthase kinase*-3β (GSK-3β). Supporting this idea, the depletion or inhibition of GSK-3β results in TFEB-mediated upregulation of PGC1α in muscle cells (Theeuwes et al., 2020), and exercise reduces GSK-3β activity (Aschenbach et al., 2006). TFEB translocates to the nucleus, since GSK-3β inactivation prevents its phosphorylation at Ser134 and 138, which blocks subsequent phosphorylation at Ser211 and, therefore, avoids TFEB cytoplasmic retention by 14-3-3 (Marchand et al., 2015; Li et al., 2016). Additionally, inhibition of GSK-3β may lead to AMPK activation during exercise, since GSK-3β prevents its activation through the phosphorylation of AMPKα at Thr479 (Suzuki et al., 2013).

As described above, FLCN loss in WAT results in constitutive activation of AMPK, which leads to upregulation of PGC1α and ERRα (Yan et al., 2016). Consequently, there is an increase in mitochondria biogenesis and activity, fatty acid metabolism, and adipogenesis. These metabolic changes protect mice from diet-induced obesity and increases resistance to cold exposure. Other studies have implicated TFEB and TFE3 in these processes (Wada et al., 2016; Evans et al., 2019). Hence, nuclear translocation of TFE3 by FLCN loss, or TFEB overexpression induces upregulation of PGC1β or PGC1α, respectively, in adipocytes and/or adipose tissue. This results in increased expression of genes involved in mitochondrial biogenesis and activity, and adipose tissue browning (Figure 3). These studies further support a coordinated activation of metabolic processes by TFEB/TFE3 and PGC cofactors and suggest a role of AMPK as a key upstream regulator of these factors.

TFE3 overexpression enhances PGC1α expression in hepatocytes (Xiong et al., 2016). In these cells, TFE3 prevents steatosis by promoting autophagy-induced lipophagy and PGC1α-mediated fatty acid β-oxidation (Figure 3). Similarly, TFEB controls lipid metabolism through PGC1α, a mechanism conserved in *C. elegans* (Settembre et al., 2013).

Other examples of interplay between TFEB/TFE3 and PGC1α are found in the central nervous system (CNS). The role of these factors as regulator of lysosomal and mitochondrial activity as well as autophagy may be necessary to prevent neurodegenerative disorders such as Parkinson’s (PD) and Huntington’s diseases (HD). Parkin is a ubiquitin E3 ligase that promotes the degradation of damaged mitochondria by mitophagy. Mutations in *PARK2*, Parkin gene, lead to mitochondrial dysfunction, a hallmark of Parkinson’s disease (Kitada et al., 1998). A Parkin Q311X mutant mice presents defective mitochondrial function and other characteristics of PD such as α-synuclein aggregates and loss of DAergic SNpc cells. Molecular characterization of this mutant showed that Q311X mutation results in the accumulation of PARIS, a PGC1α transcriptional repressor (Shin et al., 2011), and, therefore, reduced levels of PGC1α and TFEB (Figure 3; Siddiqui et al., 2015). Decreased TFEB/PGC1α was associated with the phenotypic characteristics of this mutant. Hence, rapamycin treatment or TFEB upregulation rescue all the phenotypic alterations (Decressac et al., 2013; Siddiqui et al., 2015). Huntington’s disease is caused by the misfolding and aggregation of a form of huntingtin protein with an expanded polyglutamine tract at its N-terminal region. This form of huntingtin represses transcription of PGC1α (Cui et al., 2006), which correlates with reduced expression of TFEB in a mouse HD model (Tsunemi et al., 2012). Overexpression of PGC1α induces transcription of TFEB and causes TFEB-dependent disaggregation of huntingtin complexes and amelioration of neurotoxicity (Figure 3). Again, these results underscore the relevant role of TFEB/TFE3-PGC1α positive feedback loop in the control of essential cellular processes and point out these factors as putative therapeutic targets.

In renal tubular cells, PGC1α-induced TFEB expression protects cells from genotoxic stress by promoting mitophagy (Figure 3; Lynch et al., 2019). Similarly, TFEB and PGC1α cooperate to protect cardiomyocytes from death after ischemia-reperfusion injury (Figure 3; Ma et al., 2015). These factors may maintain mitochondrial homeostasis and, in turn, prevent Beclin-1 upregulation. Beclin-1 stimulates mTORC1, which by blocking TFEB/PGC1α function may lead to cardiomyocyte death (Ma et al., 2015).

Taken together, these data indicate that TFEB/TFE3 and PGC1α interplay is important to maintain cellular homeostasis. Coordinated control of lysosomal function, autophagy, and mitochondrial biogenesis give the cells tools to response against different types of stresses, obesity, and protein aggregates. These results underline TFEB/TFE3 and PGC1α as potential therapeutic targets for the treatment of metabolic and neurological diseases.

#### FLCN/mTORC1/TFE3 in Stem Cells

*Folliculin*/*folliculin interacting protein 1* complex is critical for the exit from pluripotency of human and mouse *embryonic stem cells* (ESCs) (Betschinger et al., 2013; Mathieu et al., 2019). This process requires FLCN/FNIP GAP activity on Rag C/D to facilitate the binding of TFE3 to the Rags, its mTORC1-mediated phosphorylation, and subsequent retention in the cytoplasm (Mathieu et al., 2019; Villegas et al., 2019). Accordingly, FLCN-, FNIP1/2-, or Ragulator-deficient ESCs fail to exit from pluripotency. Under these conditions, TFE3 localizes to the nucleus, where it activates transcription of genes involved in the Wnt pathway, required to maintain pluripotency (Mathieu et al., 2019). Thus, inhibition of Wnt pathway rescues FLCN loss defect in exit pluripotency. FLCN/FNIP complex may also play an important role in myoblast differentiation. A recent study identified FNIP1 as a key regulator of myoblast redox homeostasis and integrity (Manford et al., 2020). Under reductive stress, E3 ubiquitin ligase CUL^FEM1B^ binds to reduced cysteines in FNIP1 and induces its degradation by the proteasome. This results in increased mitochondrial activity to preserve redox homeostasis. Although not shown, it is plausible to speculate that this response may be mediated by TFEB/TFE3/PGC1. High mitochondrial activity triggers the stabilization of FNIP1, which may contribute to antioxidant responses. Again, these results suggest a critical role of FLCN/FNIP and TFEB/TFE3/PGC in stem cell maintenance and differentiation. Conversely, FLCN is critical to maintain adult *hematopoietic stem/progenitor cells* (HSPCs) quiescence and homeostasis (Baba et al., 2016). In these cells, FLCN loss triggers excessive proliferation that leads to rapid depletion of HSPCs, disappearance of all hematopoietic cell linages, acute bone marrow failure, and death. This phenotype is associated with hyperactivation of mTORC1, since treatment with rapamycin, an mTORC1 inhibitor, reverses all these events. As expected, TFE3 localizes to the nucleus in FLCN-deficient HSPCs and contributes to excessive proliferation, although the mechanism remains elusive (Baba et al., 2016). *Drosophila* ortholog of FLCN is required for male germline stem cell maintenance (Singh et al., 2006). These results indicate that FLCN function in stem cell biology is context dependent.

Culture of ESCs in suspension promotes the formation of aggregates called *embryoid bodies* (EB). This is a model to study differentiation and the formation of lineages corresponding to all three germ layers (Leahy et al., 1999). Using this approach, AMPK was identified as a critical factor for cell fate determination during differentiation (Young et al., 2016). AMPK carries out this function through the upregulation of TFEB. Thus, TFEB depletion recapitulates the phenotype associated with AMPK loss, while overexpression of TFEB in AMPK deficient ESCs rescues cell fate determination. This process requires TFEB-mediated increased lysosomal function and activation of Wnt signaling pathway (Young et al., 2016). Related to the role of TFEB in embryonic development, deletion of TFEB in mice results in early embryonic lethality due to defects in placental vascularization (Steingrimsson et al., 1998). These results indicate that TFEB is a crucial transcription factor in cellular differentiation and development. Therefore, it would be of interest to characterize how regulation of TFEB by FLCN, AMPK, or mTORC1 affects these biological processes.

#### FLCN/mTORC1/TFE3 in Cancer

Mechanistic target of rapamycin complex 1 is a master regulator of cell growth and proliferation. Therefore, dysregulation of mTORC1 signaling has been implicated in several disorders including cancer. Although mTOR is rarely mutated, it is a downstream effector of frequently mutated oncogenic pathways including the PI3K/Akt pathway and the Ras/Raf/Mek/Erk pathway (Menon and Manning, 2008; Paquette et al., 2018; Liu and Sabatini, 2020). Accordingly, hyperactivation of mTOR signaling is observed in 80% of human cancers (Menon and Manning, 2008). Herein, we describe other mechanisms by which mTORC1 activity can be enhanced in tumors.

Folliculin is a tumor suppressor. Deletions or loss-of-function mutations in FLCN predispose BHD patients to develop renal cell cancer. Similarly, kidney specific FLCN knockout or FLCN heterozygous knockout mice develop enlarged polycystic kidneys with pre-neoplastic lesions (Baba et al., 2008; Chen et al., 2008; Hasumi et al., 2009; Hudon et al., 2010). Despite the role of FLCN as a positive modulator of mTORC1 activity, mTORC1 is hyperactivated in renal tumors from BHD patients or FLCN knockout mouse models (Baba et al., 2008; Chen et al., 2008; Hasumi et al., 2009; Hudon et al., 2010). These tumors rely on mTORC1 pathway to grow, since treatment with rapamycin reduces tumor size. To explain the activation of mTORC1 in FLCN-deficient tumors, a recent study points out TFEB as a key factor (Napolitano et al., 2020). TFEB shows a nuclear localization and activates the transcription of its target genes including Rag C and Rag D (Di Malta et al., 2017; Li et al., 2019). Although in their inactive state in the absence of FLCN, increased levels of Rag C/D are able to activate mTORC1 as determined by the phosphorylation of S6K and 4E-BP1 (Napolitano et al., 2020). Therefore, this feedback loop between TFEB and mTORC1 plays a key role in the development of FLCN-deficient renal tumors. A similar mechanism was described in salivary gland tumors from BHD patients. In this case, hyperactivation of mTORC1 is associated with increased TFE3 activity (Isono et al., 2020).

TFE3 gene translocations, and less frequently TFEB, are detected in renal cell carcinoma, alveolar soft part sarcoma, and perivascular epithelioid cell neoplasm (Perera et al., 2019). The fusion of *TFE3* gene to strong promoters such as *PRCC*, *ASPSCR1*, *SFPQ*, *NONO*, or *CLTC*, or fusion of *TFEB* gene to MALAT1 promoter has been identified in these tumors. Resulting fusion proteins lack their Rag binding site, therefore, bypass mTORC1-mediated surveillance, and translocate to the nucleus, where they activate their transcriptional program to promote cancer cell proliferation and survival (Kauffman et al., 2014; Yin et al., 2019). Multiple molecular pathways may be regulated by fusion TFEB/TFE3 proteins, including mTORC1 (Argani et al., 2010b; Kauffman et al., 2014; Damayanti et al., 2018). These results suggest a critical role of mTORC1-TFEB/TFE3 feedback loop in these tumors too. Accordingly, mTORC1 inhibition or silencing of TFE3 or Rag D reduces mTORC1 activity and proliferation of renal cancer cells expressing TFE3 fusions (Di Malta et al., 2017; Damayanti et al., 2018). In these cellular setting, hyperactivation of mTORC1 may be promoted by TFEB/TFE3-mediated upregulation of FLCN and FNIP1/2 in addition to Rag C/D (Martina et al., 2014; Di Malta et al., 2017).

Activation of TFEB and TFE3 is observed in other cancer types such as lung, pancreatic ductal adenocarcinoma (PDA), breast, prostate, and colorectal, but it is not associated with chromosomal translocations or FLCN mutations (Liang et al., 2018; Torres et al., 2018; Perera et al., 2019). In PDA cell lines and patient samples, MiTF-TFE proteins are upregulated and their transcriptional activity is required to keep high levels of lysosomal function and autophagy (Perera et al., 2015). Nuclear localization of MiTF/TFE factors is mediated by Importin 7 and 8, nucleocytoplasmic transporters that are overexpressed in these cancer cells and tumors. Increased lysosomal activity is necessary to maintain intracellular levels of amino acids and is essential for tumor growth. Amino acid availability, and, probably, upregulation of Rag C/D and FLCN/FNIP promote mTORC1 activation and anabolic metabolism, while lysosomal function and autophagy allow rapid adaptation to stress conditions (Perera et al., 2015). TFE3-induced elevated lysosomal activity is also required for tumor growth in a murine model of Kras^G12D^ *non-small-cell-lung cancer* (NSCLC). In this case, AMPK activation is responsible for the induction of TFE3 function (Eichner et al., 2019). All these results highlight an important role of mTORC1/TFEB/TFE3 feedback loop in cancer biology and encourage to better-characterize how mTORC1-FLCN/FNIP-AMPK interplay to regulate TFEB/TFE3 activity.

#### FLCN/AMPK/PGC1α in Cancer

Cancer cells have the capacity to rewire their metabolism to produce the energy required for tumorigenesis, tumor progression, and metastasis (Pavlova and Thompson, 2016; Guo et al., 2020). A hallmark of cancer cells is the “Warburg effect”, which implies a switch from oxidative phosphorylation to aerobic glycolysis (Hanahan and Weinberg, 2011; Faubert et al., 2013). AMPK activity affects the “Warburg effect” in opposite ways depending on the cellular context. In hematopoietic cells, AMPK has a negative effect, since AMPK loss leads to mTORC1-mediated translational upregulation of *hypoxia-inducible transcription factor*-1α (HIF1α), a master regulator of the “Warburg effect” (Faubert et al., 2013; Vara-Ciruelos et al., 2019). In contrast, other studies support a positive role of AMPK on glucose uptake and aerobic glycolysis. Thus, our group observed that activation of AMPK by FLCN loss leads to upregulation of PGC1α and, consequently, elevated mitochondrial biogenesis and oxidative phosphorylation in cancer cells (Yan et al., 2014). This results in the accumulation of *reactive oxygen species* (ROS) that activate HIF1α and promote aerobic glycolysis (Jung et al., 2008; Yan et al., 2014). These metabolic rearrangements confer tumorigenic advantages to FLCN-deficient cancer cells. Supporting these observations, activation of AMPK and upregulation of PGC1α was detected by immunohistochemistry in BHD-derived renal tumors. In these tumors, increased mitochondrial biogenesis and metabolism were associated with upregulation of PGC1α/*mitochondrial transcriptional factor A* (TFAM) transcriptional activity (Klomp et al., 2010), and lead to their hyperplastic phenotype (Hasumi et al., 2012). AMPK-mediated upregulation of PGC1α and metabolic rewiring have been implicated in the proliferation of prostate cancer cells (Tennakoon et al., 2014). Although oncogenic function of AMPK/PGC1α axis is not extensively reported, the contribution of AMPK and PGC1α to tumor development and progression have been clearly established. Further studies may confirm the relevant role of AMPK/PGC1α in cancer biology.

## Other Functions of FLCN

**FIGURE 4:**
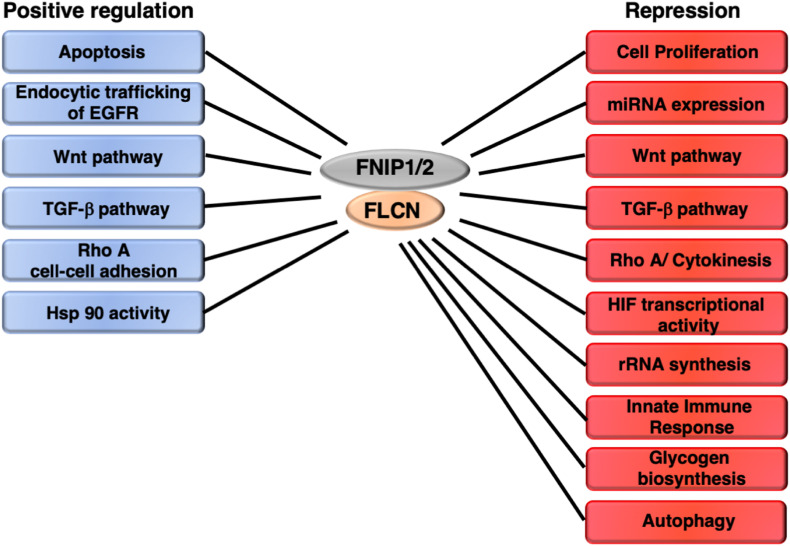
Other pathways and cellular processes regulated by FLCN/FNIP independently of AMPK and mTORC1. FLCN shows an opposing effect on certain pathways depending on the cellular context.

Folliculin has been implicated in the regulation of other cellular processes and signaling pathways. However, there is not enough evidence to support a role of FLCN as a modulator of AMPK and/or mTORC1 in these events. These processes include rRNA biogenesis, miRNA regulation, WNT, TGF-β, EGFR, Ras, RhoA, and HIF1α signaling pathways, cell proliferation, apoptosis, immune and stress responses, and Hsp90 activity (Figure 4).

### rRNA Biogenesis and miRNA Regulation

Folliculin has been identified as a negative regulator of rRNA synthesis in *Drosophila* and human cell lines (Gaur et al., 2013). FLCN localizes in the nucleolus, where interacts with the 19S proteasomal ATPase Rpt4, a regulator of rRNA transcription, and impairs its binding to the rDNA locus. Consequently, FLCN-deficient UOK-257 cells show higher levels of pre-ribosomal RNAs and mature ribosomes (Gaur et al., 2013). Overexpression of FLCN or depletion of Rpt4 reverses this phenotype. mTORC1, which is hyperactivated in this cell line, controls rRNA synthesis through the regulation of the transcriptional activity of TIF-IA (Mayer et al., 2004; Tsang et al., 2010). Thus, FLCN loss may enhance protein synthesis by increasing ribosome levels and mTORC1 activity on the translational machinery through the regulation of S6K and 4E-BP1. These phenotype is associated with tumor development (Sulima et al., 2017) and may drive FLCN-deficient tumor growth.

Interestingly, FLCN has been related to miRNA expression in cystic lesions of *primary spontaneous pneumothorax* (PSP) in BHD patients. Specifically, upregulation of *miR-424-5p* and *let-7d-5p* expression was associated with FLCN loss in these lesions (Min et al., 2020). These microRNAs show a cell type-specific effect. Elevated levels of *miR-424-5p* lead to TGF-β-induced apoptosis in lung epithelial cells BEAS-2B, while upregulation of *miR-424-5p* and *let-7d-5p* suppresses Wnt pathway and induces *mesenchymal-to-epithelial transition* (MET) in lung fibroblast HELF. Similar effects were detected in cystic tissues of PSP-BHD patients, and may account for the lung lesions associated with this disease.

### Signaling Pathways

There are evidence supporting FLCN as a regulator of the Wnt pathway, although its impact on this pathway is cell type dependent. We already described that the Wnt pathway was upregulated in FLCN-deficient ESCs. In these cells, TFE3-induced Wnt pathway is required to keep pluripotency. In contrast, we have just mentioned the negative effect of FLCN loss on the Wnt pathway in lung fibroblast and its implications in lung lesions developed in PSP-BHD patients. Similar effect was described in other report (Kennedy et al., 2019), but, in this case, TFE3 mediates the inhibition of the Wnt pathway.

As discussed above, TGF-β signaling pathway is also affected by FLCN. TGF-β signaling is involved in cell proliferation and differentiation, apoptosis, immune regulation, angiogenesis, adhesion, and migration (Roane et al., 2019). In tumorigenesis, TGF-β has a dual role. It inhibits tumor cell growth at early stages but promotes cell growth in the late phase of tumorigenesis. The effect of FLCN on TGF-β signaling is diverse. In some studies, FLCN loss leads to downregulation of genes involved in the TGF-β pathway such as *TGF-*β*2, INHBA, SMAD3* and *THBS1*, which was proposed to prevent TGF-β tumor suppressor function (Hong et al., 2010b; Cash et al., 2011). In other report, activation of this pathway was associated with tumor growth in a kidney-specific FLCN-deficient mouse model (Chen et al., 2015).

Folliculin is a GAP for Rag C/D (Tsun et al., 2013; Lawrence et al., 2019; Shen et al., 2019) and Rab7A, a member of the Rab family of small GTPases. Rab7A is important for the endocytic trafficking and lysosomal degradation of plasma membrane receptors such as EGFR. Thus, FLCN depletion slow trafficking of EGFR from early to late endosomes and enhances activation of EGFR upon binding to its ligand, which is rescued by Rab7A overexpression (Laviolette et al., 2017). Increased EGFR activity is observed in renal tumors from BHD patients and in a murine model of BHD-related kidney cancer. Inhibition of EGFR with afatinib decreases tumor growth in mice, suggesting an important contribution of EGFR signaling to the progression of BHD-associated renal tumors. Expression of Rab27B, other member of the Rab family, is negatively regulated by FLCN in follicular thyroid carcinoma cells (Reiman et al., 2012). Although Rab27B has been involved in tumor progression, metastasis, and drug resistance, the effect of FLCN-mediated regulation on its oncogenic activity is not known.

Other small GTPases such as *Ras homolog gene family, member A* (RhoA) are regulated by FLCN. RhoA signaling pathway controls cell growth, transformation, and cytoskeleton dynamics. FLCN interacts with p0071 (PKP4/plakophilin), an armadillo repeat-containing protein that binds to Rho A and modulates Rho A function in cytokinesis (Medvetz et al., 2012; Nahorski et al., 2012). p0071 also localizes in cell junctions and plays a key role in cell-cell adhesion. FLCN effect on Rho A is controversial. In one study, FLCN loss leads to deregulation of Rho A, which results in defects in cytokinesis and increased number of multinucleated cells. Additionally, FLCN-deficient FTC-133 thyroid cancer cells show higher migration capacity (Nahorski et al., 2012). FLCN expression or inhibition of Rho A signaling reverses this phenotype. In contrast, decreased Rho A activity was linked to FLCN loss in other study (Medvetz et al., 2012). In this case, elevated cell-cell adhesions and reduced cell migration in a wound healing assay were associated with FLCN depletion.

Hypoxia-inducible transcription factor-1 is other transcriptional factor whose activity increases in the absence of FLCN. High activity, but not levels, of HIF1 is observed in BHD-derived renal tumor cell line UOK257 and in renal carcinomas from BHD patients (Preston et al., 2011; Yan et al., 2014). Concomitantly, overexpression of HIF target genes (*VEGF, BMP3* and *CCND1*) and increased HIF-induced glycolytic metabolism are observed. Regulation of HIF by FLCN may occur through mTORC1 and/or AMPK. Thus, inhibition of mTORC1 with rapamycin suppresses upregulation of HIF target genes under hypoxic conditions (Preston et al., 2011). Under normoxia, AMPK activation induces the expression of PGC1α, which enhances mitochondrial biogenesis and metabolism in FLCN-deficient cells. As a result, increased ROS production induces HIF1 transcriptional activity and metabolic reprogramming (Yan et al., 2014). Control of HIF1 by FLCN has been reported in *C. elegans* too. *Flcn-1* deficiency in worms extends lifespan, which is HIF1-dependent (Gharbi et al., 2013). However, neither HIF1 activity nor HIF1 target genes expression were upregulated in *flcn-1* mutants, supporting a role of FLCN as a modulator instead of an activator of HIF1.

### Proliferation and Apoptosis

Folliculin activity has been linked to dynamic progression of cell cycle. Specifically, FLCN loss accelerates the progression through G2/M phase in UOK257 cells (Laviolette et al., 2013). However, how this process is regulated by FLCN need to be elucidated. Some evidence suggests that FLCN may control cell cycle progression through the regulation of Cyclin D1 expression. It was proposed that FLCN promotes *CCDN1* mRNA degradation, but this mechanism needs to be validated (Kawai et al., 2013). It is well-known that mTORC1 activation enhances translation of *CCDN1* mRNA (Rosenwald et al., 1993). Therefore, FLCN depletion might increase Cyclin D1 levels by stabilizing its mRNA and enhancing its translation in certain cellular context. As an oncogene (Velasco-Velazquez et al., 2011), elevated levels of Cyclin D1 may contribute to BHD-related tumor progression. Defects in cell division has been observed in FLCN-deficient *Drosophila* too (Liu et al., 2013). In zebrafish, FLCN is important for embryonic brain development. In absence of FLCN, the numbers of cells in G1 increase, while cells in S-M phase are reduced in embryonic brain (Kenyon et al., 2016). These results suggest an important role of FLCN in embryogenesis in zebrafish, as was previously described in mice (Hasumi et al., 2009).

Along this review, we have described several examples in which FLCN loss impairs apoptosis. Reduced TGF-β signaling associated with FLCN deficiency results in low levels of the pro-apoptotic factor Bim, which protects BHD-derived cells and tumor from apoptosis (Cash et al., 2011). Other pro-apoptotic proteins such as CASP1, Smac/Diablo, and HtrA2/OMI are poorly expressed in the absence of FLCN (Reiman et al., 2012). FLCN depletion protects from apoptosis by AMPK-mediated activation of autophagy in *C. elegans* and MEFs (Possik et al., 2014, 2015).

### Autophagy

Autophagy is a process by which damaged organelles and macromolecules are degraded and recycled to provide the energy and building blocks necessary to maintain cellular homeostasis and survival under stress conditions. Evidence supporting the implication of FLCN in the regulation of this process has been reported. Studies from our group identified FLCN as a negative regulator of AMPK in *C. elegans* and mammalian cells (Possik et al., 2014, 2015). AMPK directly induces autophagy through the phosphorylation of autophagy proteins such as ULK-1, VPS-34, and BECN1 (Egan et al., 2011; Kim et al., 2011, 2013) and probably by modulating TFEB/TFE3 transcriptional activity. Therefore, loss of FLCN in *C. elegans* and mammalian cells led to AMPK-mediated induction of autophagy (Possik et al., 2014). Activation of autophagy results in increased ATP levels and confers resistance to energy depleting stresses by inhibiting apoptosis. This mechanism may provide an energetic advantage to FLCN-deficient tumors and facilitate their progression under metabolic stress conditions.

### Immune Response

Some studies suggest a role of FLCN in innate immune response. Initial reports described the nuclear translocation and activation of TFEB, TFE3, and HLH-30, their ortholog in *C. elegans*, in response to bacterial infection in macrophages and worms, respectively. Consequently, the expression of proinflammatory cytokines and chemokines increases in macrophages and the expression of antimicrobial genes is elevated in worms (Visvikis et al., 2014; Pastore et al., 2016). Recent studies have implicated FLCN as a regulator of TFEB/TFE3 in this process (El-Houjeiri et al., 2019; Li et al., 2019). Thus, loss of *flcn-1* in worms, depletion of FLCN or lipopolysaccharide (LPS)-induced reduction of FLCN levels in macrophages leads to the activation of TFEB/TFE3-mediated immune response, independently of mTORC1 activity. Interestingly, our group has demonstrated that this process requires AMPK function (El-Houjeiri et al., 2019). We previously showed that loss of FLCN results in chronic activation of AMPK (Possik et al., 2014, 2015). Accordingly, simultaneous loss of *aak-1* and *aak-2*, AMPK α1/α2 orthologs in *C. elegans*, in wild-type and *flcn-1* mutant animals reduces nuclear translocation of HLH-30 and abolishes their increased survival to bacterial infection. By contrast, overexpression of a constitutively activate catalytic subunit *aak-2 oe* confers pathogen resistance. Similarly, AMPK activation with the specific activator GSK-621 in MEFs or macrophages leads to TFEB/TFE3 nuclear translocation and increased production of various cytokines and chemokines. These results support a key role of FLCN/AMPK/TFEB/TFE3 axis in innate immune response.

Folliculin and FNIP1/2 have been recently associated with the expression of interferon response genes in renal cells and BHD tumors (Glykofridis et al., 2021). Thus, deletion of FLCN or FNIP1/2 results in STAT2-mediated induction of interferon response genes, independently of interferon. How FLCN and FNIPs regulate STAT2 expression needs to be elucidated.

### Glycogen Metabolism

Glycogen biosynthesis is also controlled by FLCN. Glycogen accumulates in phagocytes from hematopoietic-linage-specific FLCN-deficient mice, the kidneys from kidney-specific FLCN-depleted mice, and renal tumors from BHD patients (Possik et al., 2015; Endoh et al., 2020). Genes involved in glycogen synthesis were upregulated in the absence of FLCN (Possik et al., 2015; Endoh et al., 2020) and their expression has been associated with TFEB/TFE3 activity in phagocytes, cardiomyocytes, skeletal muscle, and liver (Nakagawa et al., 2006; Iwasaki et al., 2012; Kim et al., 2014; Mansueto et al., 2017; Endoh et al., 2020). In worms, our group described an essential role of AMPK in glycogen accumulation in wild-type and *flcn-1* mutant strains (Possik et al., 2015), suggesting that FLCN/AMPK/TFEB/TFE3 axis is key for this process. Glycogen reserves confer resistance to hyperosmotic stress to the worms, since they can be degraded leading to the accumulation of the organic osmolyte glycerol through the activity of glycerol-3-phosphate dehydrogenase enzymes (*gpdh-1* and *gpdh-2*) under stress conditions. These results indicate that, in addition to energy reservoir, glycogen is important for other physiological processes. In fact, glycogen accumulates in many cancer types and increasing evidence points out a key role of glycogen in carcinogenesis (Possik et al., 2015; Khan et al., 2020). Thus, glycogen catabolism drives cancer cell proliferation, survival, and protection from hypoxia in models of glioblastoma, breast, and colon cancer. Glycogen catabolism is also important to overcome oxidative stress, which is important for cancer cells to survive during early steps of metastasis. Glycogen accumulation has been associated with senescence and chemoresistance. These results encourage to study other pathways by which FLCN/AMPK/TFEB/TFE3 axis may contribute to tumorigenesis.

### Hsp90 Activity

*Heat shock protein 90* (Hsp90) is a molecular chaperone responsible for the stability and activity of many substrates known as “client proteins”, including factors involved in tumorigenesis and metastasis. Hsp90 ATPase activity is required for its chaperone function and its modulated by co-chaperone. Interestingly, FLCN was identified as an Hsp90 client and FNIP1/2 as co-chaperones. Binding of FNIP1/2 to Hsp90 slows its ATPase activity, which facilitates FLCN interaction with Hsp90 and, consequently, increases FLCN stability (Woodford et al., 2016). FNIP1 function as Hsp90 co-chaperones is modulated by CK2-mediated sequential phosphorylation at Ser938, 939, 941, and 948 (Figure 1). When phosphorylated, FNIP1 binds to Hsp90 and decreases its ATPase activity. Dephosphorylation of FNIP1/2 by Ser/Thr protein phosphatase PP5 allows the addition of O-linked-N-acetylglucosamine to Ser938, which leads to its proteasome degradation (Sager et al., 2019). Intriguingly, AMPK subunits, RAPTOR, and mTOR are Hsp90 clients (Sager et al., 2018), which suggests that FNIP1/2 may additionally regulate these pathways through Hsp90 activity.

## Concluding Remarks

Folliculin was characterized as a tumor suppressor, and loss-of-function mutations were identified as the cause of the phenotypic features associated with BHD syndrome. Most of the initial studies addressed the contribution of FLCN loss to the development of BHD-derived renal tumors. From these investigations, FLCN and its partners FNIP1/2 arise as regulators of two main signaling pathways, mTORC1 and AMPK. These two pathways are critical to maintain cellular homeostasis, since they sense the nutritional and/or energetic state of the cell to activate anabolic or catabolic metabolism, respective. Consequently, FLCN/FNIP complex has emerged as an essential modulator of metabolic processes, and its dysregulation has been associated with metabolic diseases and cancer. We have started to comprehend how FLCN/FNIP complex performs this function. FLCN/FNIP complex shows GAP activity for Rag C/D, which was initially proposed to be essential for mTORC1 recruitment to lysosome and activation. However, recent studies question this role and suggest that this activity is required for the binding of the transcriptional factors TFEB and TFE3 to the Rags, where they are phosphorylated and inactivated by mTORC1. TFEB/TFE3 are master regulators of lysosomal biogenesis and function as well as autophagy. Through their interplay with transcriptional cofactors PGC1α/β, they may additionally control mitochondrial biogenesis and oxidative metabolism. FLCN depletion or nutrient starvation leads to the activation of a TFEB/TFE3-PGC feedback loop, which provides the nutrients and energy necessary for the cell to survive under adverse conditions. Additionally, FLCN/FNIP complex controls these transcriptional processes through AMPK, since FLCN deficiency results in its constitutive activation. How AMPK is upregulated has not been elucidated yet. AMPK regulates PGC at transcriptional and post-transcriptional levels. Although there are evidence supporting a role of AMPK in the control of TFEB/TFE3 activity, further studies need to be performed to confirm this notion.

Functional characterization of FLCN/FNIP complex is mostly based on experiments performed in FLCN-depleted cells or mouse models. This approach does not allow to determine whether post-translational modifications of FLCN and/or FNIP modulate their functions. Direct or indirect phosphorylation of FLCN and FNIP1/2 by mTORC1 and AMPK has been already demonstrated. Particularly, phosphorylation of FLCN at Ser62 and Ser302 by these pathways was found. These modifications show an opposite effect on the binding of FLCN to FNIP. FNIP1 is also phosphorylated by CK2 at Ser938, 939, 941, and 948, which affects its activity as a co-chaperone for Hsp90. All these studies suggest that regulation of FLCN/FNIP complex by mTORC1, AMPK, or other signaling pathways may be relevant for its activity. This is an important question that needs to be solved in the coming years. This knowledge may facilitate a better understanding of TFEB/TFE3 and PGC regulation and activity in metabolic processes. There is an increasing number of reports supporting the implication of FLCN and/or FNIP in other cellular processes independently of mTORC1 and AMPK pathways. There are still many questions to be explored in this area. In summary, we describe FLCN/FNIP as a multifunctional complex that plays a critical role in the modulation of mTORC1 and AMPK activity in metabolic processes. We focus on their downstream transcriptional effectors TFEB/TFE3, PGC1α/β, and HIF1/2α, which are master regulators of lysosomal biogenesis and activity, autophagy, mitochondrial biogenesis, and aerobic glycolysis. Deregulation of these pathways are associated with several diseases including cancer. Therefore, detailed characterization of these regulatory mechanisms will shed light on the biology of certain tumors and identify potential targets for therapeutic approaches.

## Author Contributions

JMJRR and RC wrote the manuscript. AP reviewed and edited the manuscript. All authors contributed to the article and approved the submitted version.

## Conflict of Interest

The authors declare that the research was conducted in the absence of any commercial or financial relationships that could be construed as a potential conflict of interest. The reviewer RP declared a past collaboration with one of the authors AP to the handling editor.
